# A new genus and species (*Cornucollis* gen. n. *masoalensis* sp. n.) of praying mantis from northern Madagascar (Mantodea, Iridopterygidae, Tropidomantinae)

**DOI:** 10.3897/zookeys.556.6906

**Published:** 2016-01-21

**Authors:** Sydney K. Brannoch, Gavin J. Svenson

**Affiliations:** 1 Department of Invertebrate Zoology, Cleveland Museum of Natural History, 1 Wade Oval Drive, Cleveland, Ohio, USA; 2 Department of Biology, Case Western Reserve University, 10900 Euclid Avenue, Cleveland, Ohio, USA

**Keywords:** Taxonomy, new species, Madagascar, Cornucollis masoalensis, Afrotropical

## Abstract

An examination of Malagasy specimens accessed within the Muséum national d’Histoire naturelle, Paris, France, produced a praying mantis (Insecta: Mantodea) of an undescribed genus and species. An investigation of the internal and external morphology, in addition to its collection locality, revealed that this specimen belongs to the Iridopterygidae subfamily Tropidomantinae. Furthermore, the specimen’s unique combination of characters justified the creation of a new genus. Geographic distributional records and external morphological character evidence are presented for *Cornucollis* **gen. n.** *masoalensis* **sp. n.** We provide a dichotomous key of the Tropidomantinae and Nilomantinae genera distributed within Madagascar. High-resolution images, illustrations of morphological characters, natural history information, and measurement data are presented.

## Introduction

An undetermined Malagasy praying mantis (Insecta: Mantodea) specimen was found in the material of the Muséum national d’Histoire naturelle, Paris, France. The specimen exhibits the diagnostic characters of the family Iridopterygidae (*sensu* Ehrmann 2002), and so we considered those iridopterygid genera with an Afrotropical, Indomalayan, and Australasian geographic distribution that feature an overall morphological similarity to the undescribed specimen. We compared the undescribed specimen to the genera that met our diagnostic criteria; these genera belong to the subfamilies Tropidomantinae (Stål 1877, Westwood 1889, Giglio-Tos 1915, Hebard 1920, Kaltenbach 1998, Ehrmann 2002, Svenson and Roy 2011, Roy and Svenson 2011), Nilomantinae (Werner 1907, Giglio-Tos 1915, Paulian 1957, Ehrmann 2002), and Nanomantinae (Hebard 1920 and Beier 1933). Those genera with an overall gross morphology that is markedly distinct from the undescribed specimen were excluded in our generic-level comparisons (*e.g.*, very small, dark brown and gray mantises with darkly colored wings). The undescribed specimen features sufficient distinctive characteristics that do not correspond to standing generic descriptions and thus justified the creation of a new genus and species. As this specimen features 4 posteroventral forefemoral spines, 3 discoidal forefemoral spines, and a distinct, elevated medial keel that traverses the pronotum, which are diagnostic characters for the Iridopterygidae subfamily Tropidomantinae (Giglio-Tos 1915), we place this genus with the subfamily.

## Materials and methods

### Taxonomic sampling

Specimens were gathered for comparison from the Muséum national d’Histoire naturelle, Paris, France (MNHN), the California Academy of the Sciences (CAS), the National Museum of Natural History (USNM), and the research collection of praying mantises at the Cleveland Museum of Natural History (CMNH). Specimen records indicate that these mantises are most often collected with mercury vapor light traps and canopy beating. Specimens were preserved on insect pins and kept in climate-controlled cabinets or in vials of 95% ethanol.

### Descriptive conventions and morphological characters

The generic- and species-level descriptions include diagnosis, taxonomic history, repository, and morphological character description. The morphological nomenclature followed Snodgrass (1935, 1937), La Greca (1954), Klass (1997), Wieland (2006, 2013), and Svenson (2014). Genitalic structures and other external diagnostic characters are indicated on figure illustrations and images. External morphology was compared directly with gathered Tropidomantinae, Nilomantinae, and Nanomantinae specimens and descriptions in the primary literature.

To isolate the genitalia from relaxed specimens, the abdominalia was excised at tergite seven using iris scissors and placed in an individual vial of 10% KOH that was heated in a 40 °C water bath for five minutes to clear the structures. Structures were cleaned of excess tissues with fine ocular forceps and a 000 insect pin secured within a pin vise. The male genitalia was dissected into three phallomeric lobes, which are described from a ventral and dorsal perspective.

For efficiency and ease of use, prothoracic femoral and tibial spine counts were expressed via a formula proposed by Rivera (2010) using the morphological nomenclature of Wieland (2013). The formula is separated into femoral and tibial sections, which detail the number and potential variability of discoidal (DS), anteroventral (AvS) and posteroventral (PvS) spines. As the foretibiae do not have discoidal spines, those are omitted from the latter half of the formula. Neither the femoral genicular spurs nor the tibial spur are included in the spine counts. Forefemoral spine size arrangement is represented using the letter “I, i”; majuscule (*i.e.*, “I”) represents relatively large spines, whereas the minuscule (*i.e.* “i”) represents relatively smaller spines.

*Measurements*. Eighteen measurements were obtained using a Leica M165C stereomicroscope and an IC80 HD coaxial video camera using the live measurements module of the Leica Application Suite (LAS). All measurements are presented in millimeters.

1.) *Body Length* = From central ocellus to distal terminus of body or wing (whichever is longer); 2. *Forewing Length* = From convergence of vannal veins to apex of forewings; 3.) *Hindwing Length* = From convergence of vannal veins to apex of hindwings; 4.) *Pronotum Length* = From anteromedial margin of pronotum to posteromedial margin; 5.) *Prozone Length* = From anteromedial margin of pronotum to supracoxal sulcus; 6.) *Pronotum Maximum Width* = From lateral edge of pronotum to opposing edge, across widest region; 7.) *Pronotum Maximum Width* = From lateral edge of pronotum to opposing edge, across narrowest region posterior to supracoxal sulcus; 8.) *Head Width* = From lateral edge of compound eye to opposing edge of contralateral eye, perpendicular to central axis of the head, across widest point; 9.) *Head Vertex to Clypeus* = From center of the cranial vertex to base of clypeus; 10.) *Lower Frons Width* = From lateral margin of lower frons at widest point to opposing outer margin; 11.) *Lower Frons Height* = From lower margin of unpaired, median ocellus to middle of epistomal suture; 12.) *Prothoracic Femur Length* = From most proximal margin abutting the trochanter to distal apex of genicular lobe; 13.) *Mesothoracic Femur Length* = From most proximal margin abutting the trochanter to distal terminus; 14.) *Mesothoracic Tibia Length* = From proximal bend of the tibia to distal terminus, at a point medially positioned between apical spur and cuticular outgrowth of the tibia; 15.) *Mesothoracic Tarsus Length* = From most-proximal margin of basitarsus to distal terminus of the segment before the ungues; 16.) *Metathoracic Femur Length* = From most proximal margin abutting the trochanter to distal terminus; 17.) *Metathoracic Tibia Length* = From proximal bend of the tibia to distal terminus, at a point medially positioned between apical spur and cuticular outgrowth of the tibia; 18.) *Metathoracic Tarsus Length* = From most-proximal margin of basitarsus to distal terminus of the segment before the ungues.

*Habitus images and illustrations*. High resolution images of the specimen and genitalia were captured using a Passport Storm© system (Visionary Digital™ 2012), which includes a Stackshot z-stepper, a Canon 5D SLR, macro lenses (50mm, 100mm, and MP-E 65mm), three Speedlight 580EX II flash units, and an associated computer running Canon utility and Adobe Lightroom 3.6 software. The z-stepper was controlled through Zerene Stacker 1.04 and images were processed using the P-Max protocol. Habitus and genitalia images were captured over an 18% grey card background for white balance standards. Images were processed in Adobe Photoshop CS6 Extended to adjust levels, contrast, exposure, sharpness, and to add scale bars. Minor adjustments were made using the stamp tool to correct background aberrations and to remove distracting debris. Plates were constructed using Adobe Illustrator CS6 and Adobe Photoshop CS6. Dorsal and ventral habitus images of the types, in addition to the original specimen labels, can be viewed online at http://specimens.mantodearesearch.com. Illustrations of key morphological structures were digitized in Adobe Illustrator CS5. All illustrations were produced by Rebecca Konte of the Cleveland Institute of Art.

## Results

**Figure 1.**
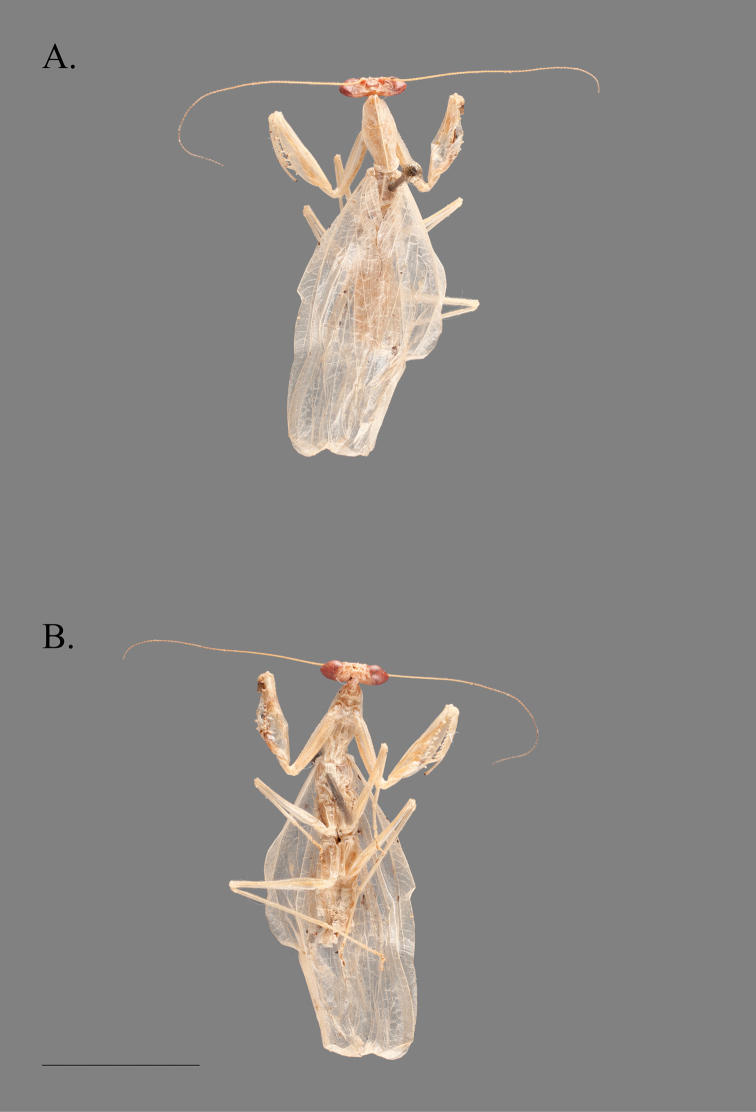
*Cornucollis masoalensis* gen.n., sp. n., male (scale bar = 10 mm). **A** dorsal habitus **B** ventral habitus.

The undescribed specimen (Fig. 1) features a unique combination of external morphological characters variously present in the Iridopterygidae subfamilies Tropidomantinae, Nilomantinae, and Nanomantinae. These features include: distinctly conical compound eyes; a concave vertex with slight juxtaocular bulges; a slightly curved ventral cervical sclerite; lateral cervical sclerite with blunted, horn-like mediolateral projection directed laterad; pronotum broad, supracoxal bulge not distinctly pronounced, lateral margins narrowly tapered in the posterior half of the metazone; pronotum with slight lateral cuticular expansions, the margins of which feature socketed setae; a distinctly elevated medial keel traversing the entire length of the pronotum; male wings opaque; foretibial posteroventral spines procumbent and approximately 15 in number. Furthermore, the male genital characters include a processo apical (**paa**) that is relatively smooth; lobo membranoso (loa) with sclerotized region of crenulation on the posterior margin; ventral sclerotization of the left phallomere complex (*i.e.*, the ventral phallomere) (L4A) without granulation, features a distinct lateral outgrowth on both the dextral and sinistral margins, the posterior margin tapering into a narrow distal process (pda); right phallomere (R1) features a right arm (bm) with a sclerotized, acuminate outgrowth. An examination of the literature as well as specimens belonging to the Afrotropical genera within each subfamily was performed to determine which genus, if any, the unidentified specimen fit within.

### Tropidomantinae

The genera belonging to the subfamily Tropidomantinae include *Tropidomantis* Stål, 1877, *Enicophlebia* Westwood, 1889, *Platycalymma* Westwood, 1889, *Hyalomantis* Giglio-Tos, 1915, *Negromantis* Giglio-Tos, 1915, *Melomantis* Giglio-Tos, 1915, *Neomantis* Giglio-Tos, 1915, *Xanthomantis* Giglio-Tos, 1915, *Kongobatha* Hebard, 1920, and *Chloromantis* Kaltenbach, 1998. While *Miromantis* Giglio-Tos, 1927 and *Oxymantis* Werner, 1931 belong to Tropidomantinae, their overall gross morphology is markedly distinct from the undescribed specimen in question and is therefore not considered here.

*Tropidomantis* differs in the following ways: compound eyes rounded; ventral cervical sclerite absent; pronotum anteriorly narrow with a distinct expansion at the supracoxal sulcus; pronotal lateral margins without distinct tapering in the posterior half of the metazone; foretibia with 8–10 posteroventral spines with sometimes the sixth spine extended; foretibial posteroventral spines relatively erect, slanted; male wings hyaline.

*Enicophlebia* differs in the following ways: compound eyes rounded; lateral cervical sclerite without mediolateral projection; pronotum with denticulate carina; foretibial spines relatively erect, slanted; forewings exceptionally broad at the base and distinctly creased proximal to the region of the pronounced pterostigma.

*Platycalymma* differs in the following ways: compound eyes rounded; cranial vertex does not retain an elevation in the shape of a truncated pyramid; lateral margins of the pronotum are smooth; medial keel of the pronotum does not traverse the length of the structure; male wings hyaline; male genital structure processo apical (**paa**) is variably granulated; male genital structure ventral sclerotization of the left phallomere complex (*i.e.*, the ventral phallomere) (**L4A**) features variable granulation, without smooth lateral outgrowths, and does not retain a narrow distal process (**pda**); male genital structure right phallomere (**R1**) features a simple right arm (**bm**) without outgrowths.

*Hyalomantis* differs in the following ways: compound eyes rounded; cranial vertical margin either straight or slightly concave medially that becomes laterally convex towards the circumocular margin; pronotum anterior without laterally expanded cuticular margins; lateral margin of the pronotum is smooth, slightly expanded in the metazone; foretibial posteroventral spines relatively erect, angled; male genital structure ventral sclerotization of the left phallomere complex (*i.e.*, the ventral phallomere) (**L4A**) without smooth lateral outgrowths and without a tapering, narrow distal process (pda); male genital structure right phallomere (**R1**) features a simple right arm (**bm**) without outgrowths.

*Negromantis* differs in the following ways: compound eyes rounded; cranial vertical margin straight or slightly higher than the compound eyes; juxtaocular bulges not retained; pronotum slender; pronotal medial keel not significantly pronounced; foretibiae thin, tubular, with 8–9 posteroventral spines.

*Melomantis* differs in the following ways: compound eyes rounded; cranial vertical margin straight with slight juxtaocular bulges; pronotum short and depressed; forewings significantly wider than the length of the pronotum, with arcuate margins.

*Neomantis* differs in the following ways: compound eyes rounded; cranial vertex higher than compound eyes; juxtaocular bulges protruding; pronotum broad with distinct expansion at the supracoxal sulcus; forewings significantly wide, wider than the length of the pronotum; foretibiae with 9–11 external spines; male forewings sub-hyaline.

*Xanthomantis* differs in the following ways: compound eyes rounded; cranial vertical margin straight or weakly concave, slightly higher than the compound eyes; pronotum relatively narrow, approximately as long as the forecoxae; supracoxal bulge distinct; metazone twice as long as the prozone; pronotal medial keel fine, extends just into the prozone; foretibial posteroventral spines of variable number and distribution.

*Kongobatha* differs in the following ways: head slightly wider than long; compound eyes rounded; cranial vertex higher than the compound eyes; juxtaocular tubercles angulate, blunted, and projecting; pronotum slender and relatively elongate, longer than the forecoxae; metazone almost twice as long as the prozone; supracoxal bulge present but not pronounced; pronotal medial keel fine; foretibiae with 9 erect, slanted posteroventral spines, the second and fourth spines proximal to the body relatively elongate; male forewings hyaline.

*Chloromantisdiffers* in the following ways: compound eyes rounded; cranial vertex straight, slightly higher than the compound eyes; juxtaocular bulges rounded and slightly projecting; pronotum short, distinctly rhombic; forewings significantly wider than the length of the pronotum; foretibiae with 8 erect, slanted posteroventral spines.

### Nilomantinae

The genera included within the subfamily Nilomantinae include *Ilomantis* Saussure 1899, *Nilomantis* Werner, 1907, *Epsomantis* Giglio-Tos, 1915, and *Mimomantis* Giglio-Tos, 1915. Additionally, we will compare Ilomantis (Saussure, 1899), which is presently the junior synonym of *Nilomantis*, for a more thorough interpretation as *Nilomantis* and *Ilomantis* have an unstable taxonomic history. *Papugalepsus* Werner, 1928 belongs to Nilomantinae but the overall gross morphology of the genus is greatly distinct from the undescribed specimen in question and is therefore not considered here.

*Ilomantis* differs in the following ways: cranial vertical margin with pronounced bulges interior to the juxtaocular bulges, which are more elevated than the juxtaocular bulges themselves; pronotum narrow with a lateral margin that is very slightly extended around the perimeter; lateral cervical sclerites do not retain a horn-like marginal projection; ventral cervical sclerite not present; anteroventral tibial spines relatively erect, slanted; male wings hyaline; male genital structure lobo membranoso (**loa**) is smooth; male genital structure ventral sclerotization of the left phallomere complex (*i.e.*, the ventral phallomere) (**L4A**) features variable granulation, without a sinistral lateral outgrowth; male genital structure right phallomere (**R1**) features a simple right arm (**bm**) without outgrowths.

*Nilomantis* differs in the following ways: cranial vertical margin straight; pronotum long and narrow with a lateral margin that is very slightly extended around the perimeter; lateral cervical sclerites do not retain a horn-like marginal projection; ventral cervical sclerite not present; anteroventral tibial spines relatively erect, slanted; male wings hyaline; male genital structure ventral sclerotization of the left phallomere complex (*i.e.*, the ventral phallomere) (**L4A**) terminates posteriorly into two lobes; male genital structure right phallomere (**R1**) features a simple right arm (**bm**) without outgrowths.

*Epsomantis* differs in the following ways: head not particularly wide; compound eyes rounded, slightly bulging; pronotal medial keel slight; forefemora with 5 posteroventral spines and 4 discoidal spines; forewings broad, costal area very broad at the base.

*Mimomantis* differs in the following ways: compound eyes rounded; cranial vertical margin straight with three tubercles on the vertex; pronotum long and narrow; pronotal medial keel does not traverse the prozone; forefemora with either 4–5 posteroventral spines (the posteroventral spine count variability is due to conflicting descriptions by Giglio-Tos (1915), Paulian (1957), and Ehrmann (2002)); foretibiae with 8–9 posteroventral spines, which are divergent basally; forewings hyaline, narrow, subparallel.

### Nanomantinae

The genera included within the subfamily Nanomantinae include *Sceptuchus* Hebard, 1920 and *Sinomantis* Beier, 1933. While *Nanomantis* Saussure, 1871, *Fulcinia* Stål, 1877, *Tylomantis* Westwood,1889, *Calofulcinia* Giglio-Tos, 1915, *Fulciniella* Giglio-Tos, 1915, *Fulciniola* Giglio-Tos, 1915, *Pilomantis* Giglio-Tos, 1915, *Ima* Tindale, 1924, *Hedigerella* Werner, 1933, *Nannofulcinia* Beier, 1965, *Machairima* Beier, 1965, and *Parananomantis* Mukherjee, 1995 belong to Nanomantinae, the overall gross morphology of these genera are greatly distinct from the undescribed specimen in question and are therefore not considered here.

*Sceptuchus* differs in the following ways: compound eyes rounded, cranial vertical margin straight, juxtaocular bulges fully rounded, not projecting; pronotum slender and relatively elongate; supracoxal bulge distinct but slight; pronotal medial keel slight, not elevated; foretibia with 7 erect, slanted posteroventral spines; male forewings narrow, hyaline.

*Sinomantis* differs in the following ways: medium-sized; compound eyes rounded; juxtaocular bulges rounded and strongly protruding; pronotum narrow and relatively long, a denticulate elevated ridge traverses the metazone between the medial keel and each lateral pronotal margin; forecoxae with denticulaton; foretibiae with 9 erect, slanted posteroventral spines; forewings sub-hyaline.

The results of these generic-level diagnostic comparisons have lead us to conclude that the combination of characters observed on the undescribed specimen is unique and does not fit any of the diagnoses of previously described Iridopterygidae genera. Subsequently, we create a new genus to place this undescribed specimen.

#### Cornucollis gen. n.

Taxon classificationAnimaliaMantodeaIridopterygidae

http://zoobank.org/EFDDB230-86BA-40FE-A684-21D43C0131DF

##### Etymology.

We name the genus for the horn-like projections that extend from the lateral cervical sclerites of the cervical region.

##### Diagnosis.

Compound eyes conical; cranial vertex concave with slight juxtaocular bulges. Ventral cervical sclerite present, arcuate; lateral cervical sclerites with blunted, horn-like mediolateral projection directed laterad. Pronotum relatively broad anteriorly but narrowly tapered in the posterior half of the metazone; pronotum with slight lateral cuticular expansions; pronotal medial keel distinctly elevated, traversing the entire length of the pronotum. Forefemora with 4 posteroventral spines and 3 discoidal spines; foretibial posteroventral spines procumbent.

##### Type species.

*Cornucollis masoalensis* sp. n. here described.

#### Cornucollis masoalensis sp. n.

Taxon classificationAnimaliaMantodeaIridopterygidae

http://zoobank.org/53024945-5633-4B20-AA94-2407DC74F0F1

##### Type.

Holotype ♂ – Madagascar, Masoala, Tampolo battage canopée, 3–XI–2001, H. Barrios & D. Randriamasimanana (Muséum national d’Histoire naturelle, Paris, France).

##### Diagnosis.

Small and relatively slender with dorsoventrally compressed cranium and likewise compressed, conical compound eyes. Pronotum length more than twice the width, relatively broad, with a slightly expanded lateral margin and deep tapering in the posterior half of the metazone; pronotal medial keel distinctly elevated, traversing the length of the pronotum. Cervical region with ventral sclerite; lateral cervical sclerite with a slightly blunted, horn-like mediolateral projection directed laterad. Anteroventral femoral spines with spineless region between distal penultimate and ultimate spine. Foretibial posteroventral spines procumbent. Forefemora = 3DS/10AvS/4PvS; Foretibiae = 12AvS/15PvS. Wings well-developed. Male genital complex with processo apical (**paa**) relatively smooth; lobo membranoso (**loa**) with sclerotized region of crenulation on the posterior margin; ventral sclerotization of the left phallomere complex (*i.e.* the ventral phallomere) (**L4A**) without granulation, with a distinct lateral outgrowth on both the dextral and sinistral margins, the posterior margin tapering into a narrow distal process (**pda**); right phallomere (**R1**) features a right arm (**bm**) with a sclerotized, acuminate outgrowth.

##### Description.

***Male*.** *Holotype*. Body length 24.22 mm; pronotum length 4.87; prozone length 1.98; pronotum width 2.18; pronotum narrow width 1.38; head width 4.22; head vertex to clypeus 1.61; frons width 1.4; frons height 0.31; prothoracic femur length 5.94; mesothoracic femur length 5.7; mesothoracic tibia length 3.38; mesothoracic tarsus length 3.022; metathoracic femur length 6.07; metathoracic tibia length 5.82; metathoracic tarsus length 4.61.

**Figure 2.**
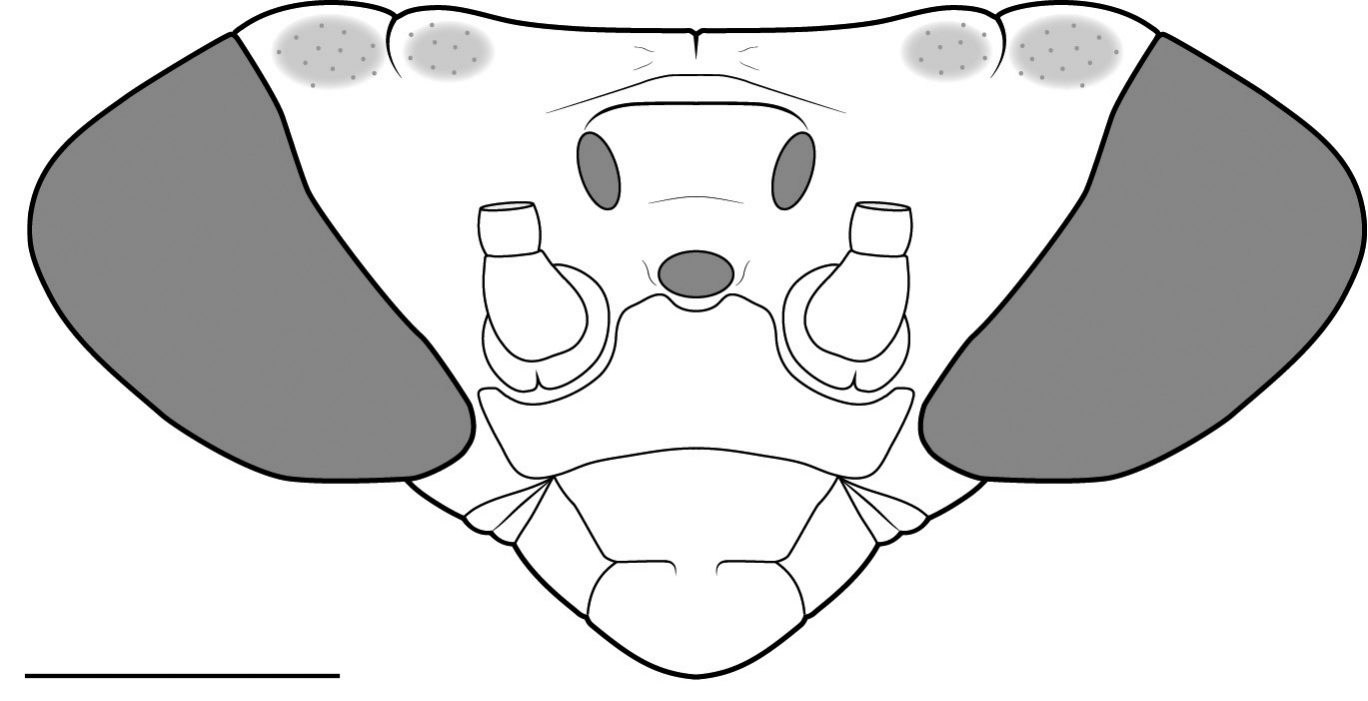
Illustration of anterior perspective of the cranium of *Cornucollis masoalensis* gen.n., sp. n., male (scale bar = 1 mm).

*Head* (Fig. 2). Patch of darkly colored speckles present on either side of the parietal sutures, near the vertical margin. Hypognathous. Juxtaocular bulges present, highlighted by parietal sutures. Head dorsoventrally compressed with likewise compressed, laterally conical compound eyes with blunted posterolateral margins. Cranial vertical margin margin is variably ciliated and cosinusoidal, the medial region strongly concave. Four gently sloping carinal ridges on the vertex (two of which originate from the mid-vertex, the other two originate from the mid-ocular region) converge into an elevation on the posteromedial vertex in the shape of a truncated pyramid, which is slightly bisected apically by the coronal suture. Vertex slightly concave posterior to the lateral, paired ocelli. Ocelli are situated atop an ocellar hill (*i.e.*, an elevated region of cuticle). Lateral, paired ocelli are larger in size, amber in color, and relatively more oblong than the unpaired, median ocellus which is relatively smaller, yellow, and approximately spherical. Lower frons transverse, the anterior margins of the structure closely abutting the posterior half of the circumantennal sclerites and the posterior half of the unpaired, median ocellus. Clypeus broad. Labrum approximately rounded along the anterior margin. Maxillary and labial palpi pale. Compound eye pigmentation darker than cuticle of the cranium. Antennae long and filiform, lightly ciliated, tapered distally.

**Figure 3.**
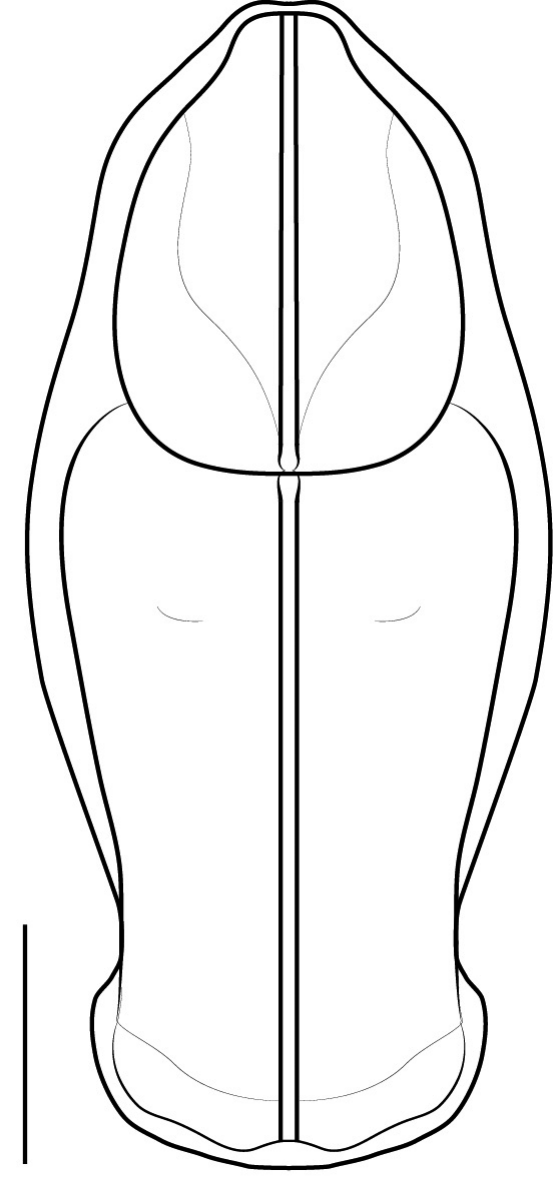
Illustration of dorsal perspective of the pronotum of *Cornucollis masoalensis* gen.n., sp. n., male (scale bar = 1 mm).

**Figure 4.**
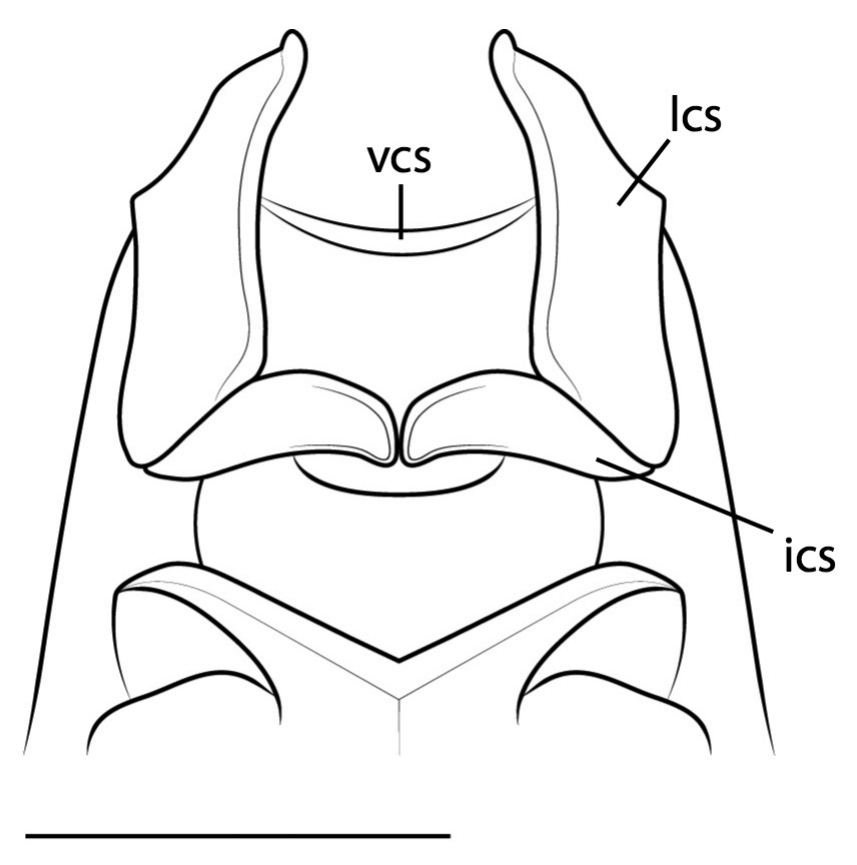
Illustration of the cervical sclerites of *Cornucollis masoalensis* gen.n., sp. n., male (scale bar = 1 mm). Abbreviations: **ics** = intercervical sclerites; **lcs** = lateral cervical sclerites; **vcs** = ventral cervical sclerite.

*Thorax*. Pronotum broad (Fig. 3); socketed setae project from perimeter. Lateral margin of the pronotum (LMP) relatively expanded around the circumference of the prozone and anterior metazone; LMP distinctly tapered in the posterior region of the metazone. Pronotal medial keel, elevated, traversing the length of pronotum. Region of pronotal medial keel is elevated, sloping down to LMP. Prozone with bilaterally symmetric sculpting, which taper to just prior to LMP expansion. Anterior metazone features a slight indentation on either side of the medial keel. Metazone posterior margin elevated into a shelf which extends slightly over the anterior margin of the mesothorax. The cervix bears lateral cervical sclerites and intercervical sclerites; one ventral cervical sclerite is present, arcuate, traversing the space between the lateral cervical sclerites (Fig. 4). The anterior portions of the lateral cervical sclerites extend just past the anterior-most region of the prozone and are lightly ciliated; Lateral cervical sclerite mediolateral margin features a distinct, horn-like acumination (Fig. 4). A furcasternal tubercle projects medially at the base of the T-shaped sclerite, posterior to the prothoracic coxae; surfaced with sternal hairs. DK hearing organ present on metathoracic ventral surface (See Yager and Svenson 2008 for hearing organ description). Wings well-developed, extending beyond base of abdominalia, opaque; relatively long cilia project along anterior portion of costal margin and relatively short cilia densely surface both the dorsal and ventral wing surface.

**Figure 5.**
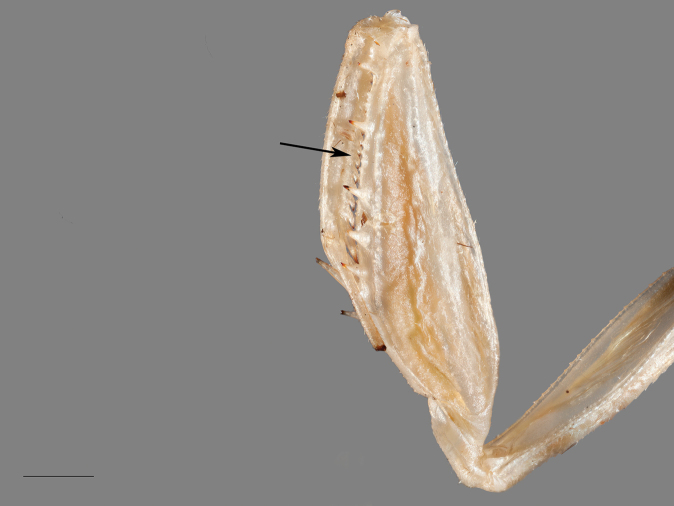
Arrow indicates procumbent posteroventral foretibial spine arrangement of *Cornucollis masoalensis* gen.n., sp. n., male (scale bar = 1 mm).

*Prothoracic legs*. Prothoracic legs are moderately surfaced with cilia and socketed setae. The forecoxae are long, extending past the base of the pronotum; postero- and anteroventral margins with socketed setae; apical lobes convergent with anterior lobe squared and posterior lobe rounded. Forefemora with a slightly arcuate dorsal margin that narrows distally. Posteroventral femoral spines robust and darkened at the apex, interspersed with cilia, socketed setae, and a row of crenulation along the posteroventral margin. Femoral genicular lobe with a moderately sized, slightly curved spine. Tibial spur groove deeply recessed, lying between first discoidal spine and the first anteroventral femoral spine. Anteroventral femoral spines alternate in size from medium to small in the following formation: IiIiIiIiII, with a spineless region between the distal penultimate and ultimate posteroventral femoral spines; spines darkened apically. The second discoidal spine is significantly longer than the first and third. Foretibiae moderately surfaced with cilia. Posteroventral tibial spines procumbent (Fig. 5); spines darkened apically; anteroventral tibial spines gradually elongate towards the tibial spur; spines darkened apically. Foretarsi unknown due to specimen damage. F= 3DS/10AvS/4PvS; T= 12AvS/15PvS.

*Meso- and metathoracic legs*. Meso- and metathoracic legs densely ciliated across surface. Posteroventral carina on the margin of the meso- and metafemora. Genicular lobes of the femora lacking spines. Tibiae tubular, featuring an apical lobe and two apical spurs. Tarsi 5-segmented with an enlarged penultimate euplantulae; darkened ungues.

*Abdomen*. Smooth, tubular, surface densely ciliated. Supraanal plate triangular, extremely narrow, ciliated; cerci long, ciliated, compressed, tapering to a point; subgenital plate terminating into two divergent rounded lobes, each featuring a short, ciliated stylus.

**Figure 6.**
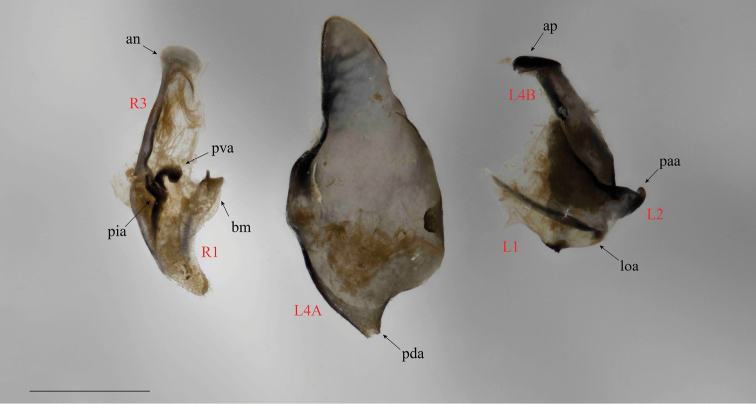
*Cornucollis masoalensis* gen.n., sp.n male genital complex. Right phallomeric lobe and dorsal sclerotization of the left phallomeric complex (*i.e.*, the left phallomere) are pictured in the ventral perspective; the ventral sclerotization of the left phallomere complex (*i.e.*, the ventral phallomere) (**L4A**) is shown in the dorsal perspective (scale bar = 1 mm). Abbreviations: **an** = anterior apodeme; **ap** = anterior process; **bm** = right arm; **L1, L2, L4B** = a sub-sclerite of the dorsal sclerotization of the left phallomeric complex; **L4A** = the ventral sclerotization of the left phallomeric complex; **loa** = lobo membranoso; **paa** = apical process; **pda** = distal process; **pia** = piastra ventrale; **pva** = processo ventrale sclerificato; **R1** = a sub-sclerite of the right phallomere; **R3** = a sub-sclerite of the right phallomere.

*Genitalia* (Fig. 6). Dorsal sclerotization of the left phallomeric complex (*i.e.*, the left phallomere) is fairly narrow anteriorly, broadening towards the posterior margin; anterior process (**ap**) of **L4B** is compact, recurved anteriorly; **ap** anterior margin heavily sclerotized; Apical process (**paa**) of **L2** is strongly dilated on the anterior margin of its visible “base,” recurved distally, narrow; **paa** with a rounded apical margin. Lobo membranoso (**loa**) relatively short, a heavily sclerotized region of crenulation projects from the posteromedial margin. Ventral sclerotization of the left phallomere complex (*i.e.*, the ventral phallomere) (**L4A**) is narrow and rounded anteriorly, with a moderately sclerotized sinistral margin; **L4A** medial sinistral margin features a distinct, broad outgrowth; **L4A** posterior region tapers dextrally into a relatively narrow distal process (**pda**); **L4A** posterodextral margin is broad; **L4A** dextral margin moderately sclerotized with a relatively small outgrowth. Anterior apodeme (**an**) of **R1** is significantly rounded anteriorly with a moderately sclerotized sinistral margin; processo ventrale sclerificato (**pva**) is strongly curved and slightly tumescent, the structure is heavily sclerotized along the posterior margin; piastra ventrale (**pia**) is relatively linear in shape with a slightly slanted anterior margin. **R1** posterior region is narrow, lightly ciliated with a tapered, rounded posterior margin; the right arm (**bm**) with a distinct anterior sclerotization that features an acuminate projection.

##### Etymology.

This species is named for the Masoala peninsula of Madagascar, the region where the specimen was collected.

##### Natural history.

Specimen was collected in June in Tampolo, Masoala, Madagascar by beating the canopy of an unknown tree.

### Key to the Malagasy Tropidomantinae and Nilomantinae Genera

**Table:** 

1	Compound eyes conical in shape, dorsoventrally compressed	**2**
–	Compound eyes rounded	**3**
2	Cranial vertex cosinusoidal with slight juxtaocular bulges; enlarged protuberances interior to the parietal suture present; ventral cervical sclerite absent	***Ilomantis* (Saussure, 1899)**
–	Cranial vertex concave with juxtaocular bulges; protuberances interior to the parietal suture not retained; ventral cervical sclerite present	***Cornucollis* gen. n.**
3	Wings well-developed with pronounced pterostigma; forewings with distinct crease around pterostigmatic region	***Enicophlebia* Westwood, 1889**
–	Wings well-developed without pronounced pterostigma; forewings without crease around pterostigmatic region	**4**
4	Cranial vertex with juxtaocular bulges; pronotum generally broad in size	**5**
–	Cranial vertex without juxtaocular bulges; pronotum generally narrow in size	**6**
5	Forecoxae equal to or less than the length of the pronotum; forewing costal region relatively narrow; the subcostal vein lies near the radial vein in the proximal half, diverging distally; female abdomen is relatively widened without lateral expansions of the tergites; supraanal plate approximately as long as wide, triangular in shape but blunted apically; darkened color patches may be present on the lower frons, foretibiae, pronotum, and forewings	***Hyalomantis* Giglio-Tos, 1915**
–	Forecoxae significantly longer than the metazone of the pronotum; forewing with broadened costal region, which is a more pronounced feature in females; the subcostal vein lies near the radial vein; female abdomen is relatively widened with lateral, acuminate expansions of the tergites; supraanal plate elongate, triangular; darkened color patches not present	***Platycalymma* Westwood, 1889**
6	Forewings narrow, margins approximately parallel	***Mimomantis* Giglio-Tos, 1915**
–	Forewings narrow with anterior margin slightly rounded	***Negromantis* Giglio-Tos, 1915**

## Conclusion

An undescribed praying mantis specimen from Madagascar was observed in the entomological collection of the Muséum national d'Histoire naturelle, Paris, France. As the specimen features the following external morphological traits (small overall body size, well-developed wings, a cranial vertex with juxtaocular tubercles, and lobeless meso- and metathoracic legs), the specimen was able to be placed among other members within the family Iridopterygidae. Due to the specimen’s external morphological characters and Afrotropical distribution, we investigated genera within the subfamilies Tropidomantinae, Nilomantinae, and Nanomantinae to determine its generic-level placement. However, further study of this insect revealed a unique combination of external and internal morphological features that are not featured in present genera. These characters include a concave cranial vertex, the presence of a ventral cervical sclerite, lateral cervical sclerites with blunted, horn-like outgrowths, procumbent posteroventral tibial spines, among others. Therefore, the new genus *Cornucollis* gen. n. was created for this undescribed species *Cornucollis masoalensis* sp. n. Further field work to uncover the undescribed female conspecific, nymphs, and oothecae, will complement the description of this new genus and species.

## Supplementary Material

XML Treatment for Cornucollis

XML Treatment for Cornucollis masoalensis
